# Transcriptional profiling of *Medicago truncatula *meristematic root cells

**DOI:** 10.1186/1471-2229-8-21

**Published:** 2008-02-27

**Authors:** Peta Holmes, Nicolas Goffard, Georg F Weiller, Barry G Rolfe, Nijat Imin

**Affiliations:** 1 ARC Centre of Excellence for Integrative Legume Research, Genomic Interactions Group, Research School of Biological Sciences, Australian National University, Canberra ACT 2601, Australia; 2 Institut Louis Malardé, GP Box 30, 98713 Papeete Tahiti, French Polynesia

## Abstract

**Background:**

The root apical meristem of crop and model legume *Medicago truncatula *is a significantly different stem cell system to that of the widely studied model plant species *Arabidopsis thaliana*. In this study we used the Affymetrix *Medicago *GeneChip^® ^to compare the transcriptomes of meristem and non-meristematic root to identify root meristem specific candidate genes.

**Results:**

Using mRNA from root meristem and non-meristem we were able to identify 324 and 363 transcripts differentially expressed from the two regions. With bioinformatics tools developed to functionally annotate the *Medicago *genome array we could identify significant changes in metabolism, signalling and the differentially expression of 55 transcription factors in meristematic and non-meristematic roots.

**Conclusion:**

This is the first comprehensive analysis of *M. truncatula *root meristem cells using this genome array. This data will facilitate the mapping of regulatory and metabolic networks involved in the open root meristem of *M. truncatula *and provides candidates for functional analysis.

## Background

The root and shoot apical meristems (RAM and SAM) are established during embryogenesis and serve as a source of stem cells for plant growth and organogenesis [1]. The RAM produces all the tissues of the primary root by a highly defined pattern of cell divisions [2]. Cells produced by the meristem, known as initials, undergo proliferative cell divisions as they are added to files of different cell types and their fate is determined by positional information [3,4]. The stem cell niche in the root is maintained by a small group of cells called the quiescent centre (QC) [5,6], the QC inhibits the division of surrounding cells and is generated and maintained by the accumulation of auxin via the PIN auxin efflux carriers; in *Arabidopsis *the genes *PLETHORA1*, *PLETHORA2*, *SCARECROW *and *SHORT ROOT *are known to be necessary for QC formation [6-9]. The interplay of auxin and cytokinin controls the size of the RAM, with the action of cytokinin implicated in controlling the exit of cells from the root meristem [10,11].

Several studies that characterise gene expression in the cells of the root meristem have been published. Studies in *Arabidopsis *have used green fluorescent protein-labelled cell types and cell sorting to characterise gene expression by microarray, for specific cell types and in different zones of root development [12-14]. A root tissue specific gene expression study has also been carried out in maize (*Zea mays*) where the proximal meristem, QC and root cap were microdissected and gene expression was measured on Affymetrix rice genome arrays [15]. However the root of model legume *Medicago truncatula *presents a notably different system for study of root development to that of *Arabidopsis thaliana *or maize. At a cellular level, the root of *M. truncatula *has a significantly different RAM to that of *Arabidopsis*. Most legume roots, unlike the *Arabidopsis *root have a basic-open root meristem [16]. The difference between open and closed meristems is significant; in the open RAM, initials are not apparent indicating possible variations in the regulation cell division and differentiation between the two types of RAM. Hamamoto *et al*. [17] have shown that roots with an open meristem produce individual living border cells and more border cells than those with a closed meristem. Border cells are important for mycorrhizal and microbial interactions including the legume-rhizobia symbiosis [18] and environmental sensing.

In terms of root organogenesis, the most obvious difference between *M. truncatula *and other model plants and is the ability of *M. truncatula *to form indeterminate root nodules in association with rhizobia. Nodulation shares several aspects of lateral root organogenesis with the advantage that it is inducible and the site of organogenesis is predictable. Root organogenesis is also inducible in *M. truncatula *in tissue culture with the addition of auxin 1-naphthaleneacetic acid (NAA) to the tissue culture media. Root formation in culture is irreversible after 7 days on NAA [19] and does not require ethylene perception [20]. Thus, the morphological differences between the *M. truncatula *root and that of other model species and interest in the species as a model for root and nodule development led us to conduct the research we present here.

## Results and discussion

### The *M. truncatula *root meristem

**Figure 1:**
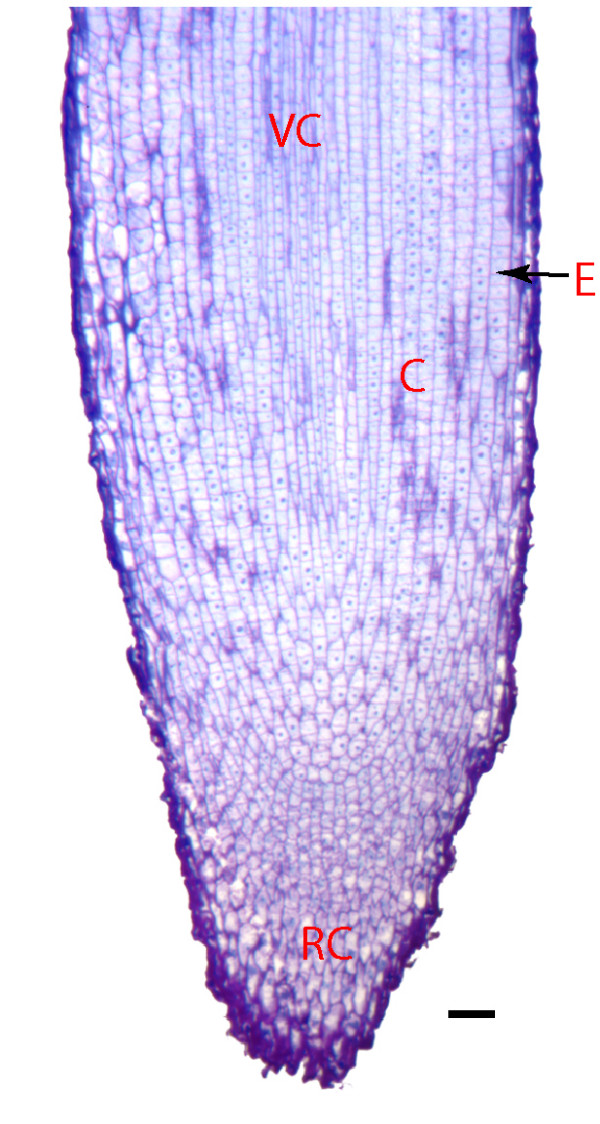
**The *Medicago *root meristem**. A median longitudinal section of the *Medicago *root stained with toluidine blue clearly shows that basic-open meristem architecture of *M. truncatula*, the zone of initials is not clearly divided into tiers; VC = vascular cylinder, C = cortex, E = epidermis, and RC = root cap; scale bar = 50 μm.

The *M. truncatula *RAM shows a characteristic basic open root meristem organisation (Figure 1). In the region where cells are dividing, it isn't possible to distinguish the initials amongst the tightly packed mass of new and elongating cells in the root tip. Our meristem section, 3 millimetres from the root tip, is comprised large group of undifferentiated cells, surrounded by some differentiated tissues including border cells, root cap and elongating cells that will form the vascular bundle, pericycle, endodermis, cortex and epidermis. Our non-meristematic section, one centimetre adjacent to the meristem, only contains the characteristic root tissue layers; root hairs occur in this region, rhizobial infection and lateral root initiation occur in this zone. These sections were chosen to correspond with earlier proteomic work on the *Medicago *root meristem [21] and because there are no described markers for specific cell types in the *Medicago *root meristem that could be used to create transgenic plants for a cell-type analysis.

To characterise the transcriptomes of the meristematic and non-meristematic root of the *M. truncatula *we extracted RNA from the meristem and non-meristematic zones from the roots of three independently grown sets of plants four days after germination. The three biological replicates were analysed using the Affymetrix *Medicago *genome array. An average of 51% (26,9610 probe sets) of the over 52, 000 plant gene probes of the Medicago Genome Array GeneChip produced 'present' calls when hybridised with biotin-labelled cRNA from *M. truncatula *roots consistent with early reports [22]. Following normalisation with GCRMA, we identified 324 transcripts that are greater that 2.0 fold over-expressed in the meristem and 363 that are over-expressed in the non-meristem. The full data set has been deposited in the Gene Expression Omnibus database as accession GSE8115; the normalised data set is available in additional file 1.

Although our meristem sample is comprised of multiple cells types, including stem cells, elongating and differentiating cells and some differentiated cell types, proteome and transcriptome data suggests that the material contains a significant proportion of stem cells. Previously we have shown with proteomic analysis that the root meristem accumulates significantly more actin and tubulin than non-meristematic tissues, consistent with cell proliferation in the meristem [21]. The array data also shows that the transcript of the ortholog of the *M. sativa *cyclin A2, *Medsa;cycA2;2 *(Mtr.44839.1.S1_at) is expressed greater than 2 fold in our meristematic root section. Cyclin A2 is important in the transition to DNA synthesis and replication phases of the cell cycle and is destroyed as the cell moves into mitosis, in *M. sativa Medsa;cycA2;2 *2 is restricted to proliferating cells designated to meristem formation during developmental programs; and expression of the gene is directly activated by auxin [23,24]. Presence of the cyclin A2 transcript at a high level therefore serves as a good indicator of stem cell activity in our root meristem section.

### Array verification

Quantitative real-time RT-PCR was used to confirm the level of expression of 10 transcripts from the array, see Table 1. The transcripts analysed were chosen based on a demonstrated or predicted role of orthologous genes in plant stem cells, or due to general interest; the functional significance of the transcripts validated by qRT-PCR is discussed in more detail below.

**Table 1:** Comparison of qRT-PCR and microarray results for selected genes

**Probe ID**	**Annotation**	**Microarray (log_2_)**	**qRT-PCR (log_2_)**
Mtr.16722.1.S1_at	DVL-like	1.84	5.80
Mtr.20966.1.S1_at	AT HOOK	0.69	3.48
Mtr.46508.1.S1_at	PLATZ	1.28	4.74
Mtr.32712.1.S1_at	LOB	0.52	3.26
Mtr.49764.1.S1_at	MtPIN9	-1.43	-2.48
Mtr.49495.1.S1_at	bHLH	1.25	4.52
Mtr.21627.1.S1_at	AP2/EREBP	1.44	4.03
Mtr.24270.1.S1_s_at	bHLH	1.52	5.18
Mtr.39218.1.S1_at	GIF	1.22	4.45
Mtr.50542.1.S1_at	GRF	1.32	6.40

The microarray data was validated using qRT-PCR. Values shown are ratios of the means of three independent measurements from microarray data or qRT-PCR.

For all probe sets, the expression ratios displayed the same pattern of expression as the array data. Low abundance transcripts that were not differentially expressed at 2.0 fold (Mtr.32712.1.S1_at and Mtr.20966.1.S1_at) were also shown to be significantly expressed by qRT-PCR. Although the qRT-PCR and microarray expression ratios were numerically different with qRT-PCR showing larger fold changes in transcript expression, the data sets were correlated with a Pearson correlation co-efficient of 0.704 (n = 10). Experimental reasons for these differences have been reviewed by Morey *et al *[25]. These results help to confirm the general accuracy of the microarray data we present here.

### Functional classification of differentially expressed probe sets

The *Medicago *genome array does not incorporate the entire *M. truncatula *genome, it was created based on an incomplete genome sequence and a ESTs from the *Medicago truncatula *Gene Index (MtGI). Over the course of the experiment we have noted the inclusion of probe sets for International *Medicago *Genome Annotation Group (IMGAG) gene predictions and the corresponding EST leading to a duplication of data, and the absence of some consensus ESTs from MtGI available at the time the chip was made (data not shown). Annotation of the probe sets on the Genome array also varies widely in quality.

**Figure 2:**
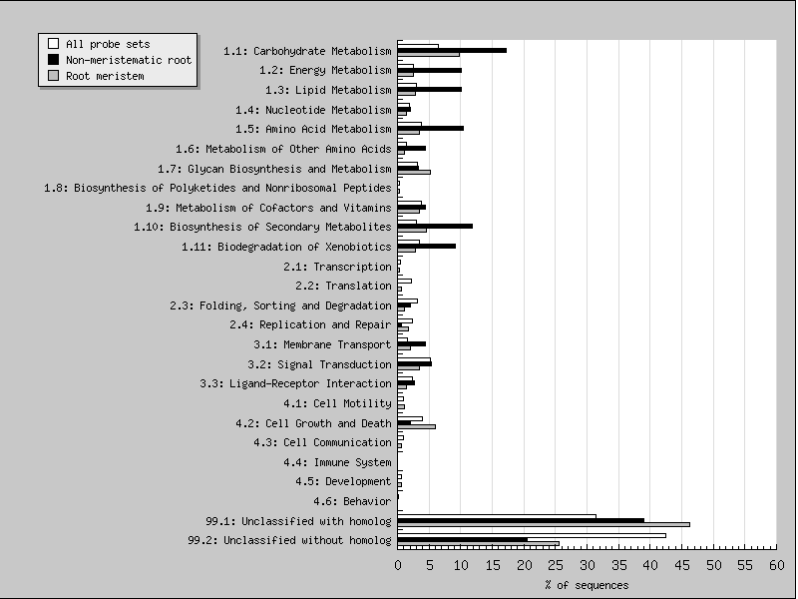
**Classification of expression changes with GeneBins**. GeneBins classification of probe sets with changes in expression that are significant at 2.0 fold.

To interpret gene expression results, we used GeneBins to assign a relationship the genes differentially expressed transcripts on the *Medicago *genome array to a hierarchical functional classification modelled on KEGG ontology [26,27]. This analysis showed that the metabolism of the root meristem and non-meristem varies significantly between the two sections, see Figure 2. About 28% percent of differentially expressed probe sets could be assigned a functional classification with GeneBins; of note 7% and 3.3% of transcripts differentially expressed are involved in carbohydrate metabolism and the biosynthesis of secondary metabolites respectively. 25.5% of differentially expressed transcripts have no homolog, however by far the largest class of probe sets that had significantly altered expression in our analysis were unclassified with a homolog. This result led us to use other bioinformatics strategies to annotate the probe sets on the genome array.

To further refine the functional classification and annotation of metabolic probe sets on the *Medicago *genome array we used PathExpress [28]. Using this database we were able to identify statistically significant over-representation of metabolic pathways in the meristematic and non-meristematic root, shown in Table 2. Four metabolic pathways are significantly over-represented in the meristem and 10 are over-represented in the non-meristematic root.

**Table 2:** Metabolic differences in meristem and non-meristematic root

**Root meristem**			
Pathway	E.C numbers in genome array pathway	No. E.C. numbers expressed >2.0 fold	P

Starch and sucrose metabolism	33	6	1.36E-03
Stilbene, coumarine and lignin biosynthesis	10	3	6.02E-03
Pentose and glucuronate interconversions	14	3	1.63E-02
**Non-meristematic root**			

Carbon fixation	22	6	2.14E-03
Lipopolysaccharide biosynthesis	10	4	2.75E-03
Gamma-Hexachlorocyclohexane degradation	9	4	1.74E-03
1,4-Dichlorobenzene degradation	8	3	1.24E-02
Flavonoid biosynthesis	14	4	1.07E-02
Penicillins & cephalosporins biosynthesis	3	2	1.26E-02
Stilbene, coumarine & lignin biosynthesis	10	3	2.42E-02
Ascorbate and aldarate metabolism	10	3	2.42E-02
Histidine metabolism	18	4	2.69E-02
Indole and ipecac alkaloid biosynthesis	5	2	3.85E-02

Metabolic pathways significantly over-represented (p ≤ 0.05) amongst differentially expressed probe sets at 2.0 fold as determined by PathExpress.

We also annotated the chip by comparing the data set with the Arabidopsis Gene Family Information database maintained by the Arabidopsis Information Resource [29]. As of April 2007 the database contained 996 gene families and 8,331 genes. Using BLAST, we were able to classify 3159 *Medicago *probe sets into these families. Sixty-nine and 71 of the differentially expressed probe sets from the meristem and non-meristem respectively were classified in the gene families; no families were significantly over-represented in either section (additional file 2). Finally, transcription factors (TF) on the Genome array were predicted by homology relationship based on the Database of Arabidopsis Transcription Factors (DATF) [30]. This analysis showed that 2932 probe sets on the Genome array have sequence homology to described plant TFs (additional file 3).

### Carbohydrate metabolism and cell wall biosynthesis

The most notable metabolic difference between the meristem and non-meristematic root is carbon metabolism, carbon is fixed in the non-meristematic root and sugars are metabolised in the meristem. PathExpress shows that transcripts of enzymes from the pathway of carbon metabolism are significantly over-expressed in the non-meristematic root; they include glyceraldehyde-3-phosphate dehydrogenase (NADP+) (Mtr.22603.1.S1_at, Mtr.52116.1.S1_at, Mtr.47901.1.S1_x_at), fructose 1,6-diphosphate phosphatase (Mtr.37533.1.S1_at), sedoheptulose 1,7-diphosphatase (Mtr.40432.1.S1_at), RuBisCO small subunit (Mtr.12203.1.S1_at, Mtr.19517.1.S1_at, Mtr.12202.1.S1_at, Mtr.19516.1.S1_at), 5-phosphoribulose kinase (Mtr.10464.1.S1_at) and phosphoenolpyruvate carboxylase (Mtr.8683.1.S1_at). PathExpress analysis also shows that transcripts of sugar metabolism enzymes are over-expressed in the meristem, they include ADP glucose pyrophosphorylase (Mtr.22751.1.S1_at), cellulose 1,4-beta-cellobiosidase (Mtr.45092.1.S1_at), beta-glucosidase (Mtr.35316.1.S1_at), cellulose synthase (UDP-forming) (Mtr.5728.1.S1_at, Mtr.35544.1.S1_at), polygalacturonase (Mtr.45925.1.S1_s_at, Mtr.18904.1.S1_at), pectinesterase (Mtr.28556.1.S1_at, Mtr.4467.1.S1_at), glucan endo-1,3-beta-D-glucosidase (Mtr.18873.1.S1_at, Mtr.44762.1.S1_s_at, Mtr.13666.1.S1_at, Mtr.44304.1.S1_at), diphosphoinositol-polyphosphate diphosphatase (Mtr.43946.1.S1_at).

Beyond basic cellular energy needs, at least two processes in the root meristem have significant energy requirements. Gravitropism requires the accumulation of starch in the root cap, a component of our root meristem sample, in organelles known as statocytes. *Arabidopsis *plants that lack or have reduced accumulation of starch in the root have reduced response to gravity [31]; gravity signalling by statocytes and other signal transduction pathways leads the redistribution of auxin in the root cap in response to gravity [32]. Another sink for sugar metabolised in the meristem is cell wall biosynthesis and modification, for a recent review of the biosynthesis of plant cell wall polysaccharides see Lerouxel *et al*. [33]. PathExpress analysis shows that enzymes implicated in the biosynthesis of stilbene, coumarine and lignin are significantly over-represented in both root sections; however in both instances the enzymes implicated are multiple isoforms of heme peroxidase, cytochrome P450 containing monooxygenases and beta-glucosidase. These enzymes contain common catalytic domains and could be involved in numerous cellular processes where reactive oxygen species are generated and detoxified, such as ascorbate and aldarate metabolism and pentose and glucuronate interconversions where they also are over-represented in the non-meristematic root in the PathExpress classification. More specifically related to cell wall biosynthesis cellulose synthase is up-regulated in the meristematic root. Cell wall plasticity is also required in dividing and elongating cells [34,35], we also find transcripts of cell wall modifying pectinesterases, polygalacturonases and expansins (Mtr.20976.1.S1_at, Mtr.22752.1.S1_s_at, Mtr.47780.1.S1_at, Mtr.9830.1.S1_at) are significantly more abundant in the meristematic root.

Neither RAM, nor SAM are photosynthetic, thus their status and carbohydrate sinks is notable. Pien *et al *[36] have linked carbohydrate metabolism with the earliest phase of commitment by meristem cells to form a leaf. They showed that meristem cells express ADP glucose pyrophosphorylase transcripts and accumulate starch with an increased frequency in the region of cells forming the leaf primordium. Based on their data they also propose that sugars may regulate the expression of genes within the meristem which encode enzymes that can function to influence sugar metabolism. Our meristem transcript data shows that expression of ADP glucose pyrophosphorylase over 2 fold and sucrose synthase over 1.5 fold. Data from *gus*-sucrose synthase reporter in *M. truncatula *demonstrates the expression of sucrose synthase in the root and nodule meristems and in cells activated to divide through association with rhizobia and endomycorrhiza [37]. The role of sugar in the modification of gene expression and its relationship with auxin in the RAM may be worthy of further investigation.

### Flavonoids

Flavonoids are important for some aspects of root and nodule development in *M. truncatula*, the analysis of an RNAi-knockdown of chalcone synthase, the enzyme that catalyzes the first committed step of the flavonoid pathway, showed that the plant can maintain active root and lateral root meristems in the absence of endogenous flavonoids but cannot initiate nodules [38]. This work also showed that flavonoid-deficient roots have an increased rate of polar auxin transport (PAT) and implicated flavonoids as a regulator of auxin transport, consistent with their reported role as endogenous auxin transport inhibitors [39,40].

Our data suggests a role for flavonoids and their derivatives in the non-meristematic root, where PathExpress shows that the flavonoid biosynthesis pathway is significantly over-represented. Isoflavone reductase (Mtr.24228.1.S1_at) is greater than 2.0 fold over-expressed in the non-meristematic root, where lateral roots are formed and symbiosis may be established with rhizobia; we have also shown the significant accumulation of this protein in the non-meristem [21]. Isoflavones have been shown to inhibit root formation *in vitro *in *M. truncatula *[19], their production is induced during nitrogen deficiency [41], and they are required for the establishment of symbiosis with rhizobia [42]. Flavonoid 3', 5'-hydroxylase (Mtr.44207.1.S1_at) and dihydrokaempferol 4-reductase (Mtr.31382.1.S1_at) both contribute to the production of anthocyanins and are also highly expressed in the non-meristematic root. Analysis of the *anthocyanninless2 *mutant of *Arabidopsis *implicates the tissue-specific accumulation of anthocyanins in sub epidermal tissues of the root in the maintenance of root organisation [43]. The relationship between anthocyanin deposition and polar auxin transport in the root has not been tested.

### Hormones and cell to cell communication

In our analysis of plant gene families we could identify probe sets that indicated that there are significant differences in cell to cell communication and response to hormones in the meristem and non-meristematic root. Auxin transport in the RAM is by a system of auxin efflux carrier proteins from the PIN family, has been well described in *M. truncatula *[44]. PIN proteins are localised in an asymmetric distribution on either the basal or apical side of cells where they control PAT [45], their expression and reorganisation is also essential for creating localised auxin gradients which are required for many aspects of plant development. In the *M. truncatula *root, *MtPIN2 *is expressed in the root, our data also showed the expression of this gene (Mtr.45124.1.S1_at), specifically up-regulated in the root meristem, consistent with the reported localisation of the AtPIN2 protein, where its localisation directs auxin flow from the root tip into the elongation zone and is crucial in mediating the gravitropic response [46]. Our array data, confirmed by qRT-PCR, also shows that *MtPIN9 *(Mtr.49764.1.S1_at) is preferentially expressed in the non-meristematic root. Multidrug resistance/P-glycoprotein-type ABC transporters also function as auxin efflux carriers, our data shows the over-expression of three homologous transcripts (Mtr.23681.1.S1_x_at, Mtr.23679.1.S1_x_at, Mtr.43342.1.S1_at) in the non-meristem with strong *C*-terminal protein sequence similarity with *Arabidopsis Multidrug Resistance-Like1 *(*MDR1*) and *MDR4*, two transporters recently shown to mediate acropetal and basipetal auxin transport proximal to the meristem respectively [47].

Cytokinin also contributes to the establishment and maintenance of the RAM, and recently it has been shown that cytokinin controls the rate of meristematic cell differentiation determining the size of the RAM through a two-component receptor histidine kinase-transcription factor signalling pathway [48]. In the RAM cytokinin is detected by *ARABIDOPSIS *HISTIDINE KINASE 3, which leads to the expression of *ARABIDOPSIS RESPONSE REGULATOR *transcription factors. In our root meristem section we were able to detect the accumulation of a probe set orthologous to *ARABIDOPSIS HISTIDINE KINASE 5 *(Mtr.30157.1.S1_at); *AHK5 *has been shown to be expressed in the elongating root where it acts as a negative regulator in the signaling pathway in which ethylene and abscisic acid inhibit root elongation through ethylene receptor ETR1 [49].

Receptor-like kinases (RLKs) have been implicated in numerous developmental signalling pathways in plant development. We see significant differential expression of three RLKs in the meristem (Mtr.5784.1.S1_at, Mtr.1137.1.S1_s_at, Mtr.413.1.S1_at) and one in the non-meristem (Mtr.50901.1.S1_at). Mtr.1137.1.S1_s_at is an ortholog of *CLAVATA1*, the leucine-rich RLK responsible for specification of the SAM through interaction with the CLAVATA3 peptide. A CLAVATA-like pathway has been implicated in RAM maintenance, root specific RLKs have been identified in *Arabidopsis *with the peptide CLAVATA3/ESR-RELATED 19 (CLE19) a possible ligand, but due to redundancy within this large family of kinases the pathway has not yet been fully described [50,51].

In the microarray data we identified several putative peptide hormone transcript highly expressed in the root meristem. The probe set (Mtr.16722.1.S1_at), expression confirmed by qRT-PCR (Table 1) has strong protein sequence similarity to the *Arabidopsis *gene *DEVIL 19*, a member of the DVL gene family. Some DVL peptides have been in shown to inhibit cell proliferation during leaf development [52,53], their receptor is unknown. We also find transcripts homologous to Rapid Alkalization Factors (RALF) expressed highly in the meristem (Mtr.18300.1.S1_at and Mtr.35639.1.S1_s_at); RALFs have been shown to act as peptide hormones in tobacco and *Arabidopsis *[54]. These peptides may have a role in *M. truncatula *meristem maintenance.

### Transcription factors

Of the 2,957 probe sets on the genome array have sequence homology to described plant TFs, 37 predicted TFs were up-regulated in meristem and 18 TFs were up-regulated at least 2 fold in non-meristematic cells (Additional file 3). Of the 64 predicted TF families in the DATF database, only 21 were differentially expressed in meristem and non-meristematic root (Table 3). Of these, nine families were significantly over-represented (p ≤ 0.05) within the up-regulated probe sets, no TF families were over-represented in the non-meristem. The families up-regulated in the meristem are the basic/helix-loop-helix (bHLH), basic leucine zipper (bZIP), growth regulating factor (GRF) and the GRF-interacting factors (GIF), APETALA2 and ethylene-responsive element binding proteins (AP2/EREBP), auxin-responsive protein/indoleacetic acid-induced protein (AUX/IAA), GATA factors (C2C2-GATA), auxin-response factors (ARF) and plant AT-rich sequence- and zinc-binding proteins (PLATZ). With the exception of bHLH, bZIP and C2C2-GATA domain containing TFs, the significantly up-regulated TF gene families are plant specific. We confirmed the expression of several TFs that significantly accumulate in the meristem using qRT-PCR (Table 1).

**Table 3:** Transcription factors

	Number of probe sets		
Family	On array	> 2 fold over-expressed in meristem	>2 fold over-expressed in non-meristem

AP2/EREBP	140	5	
ARF	48	2	
bHLH	277	10	1
bZIP	90	3	1
C2C2-DOF	274	1	
C2C2-GATA	28	2	
C3H	169		1
C2H2	199		1
CCAAT-HAP3	12	1	1
GARP-G2-like	48	1	1
GIF	3	1	
GRAS	75		2
GRF	8	2	
HB	104	1	2
HSF	72	2	
MYB	209		1
NAC	318		1
PLATZ	8	1	
WRKY	837	4	6
ZF-HD	13	1	

Transcription factors were predicted by homology relationship based on the Database of Arabidopsis Transcription Factors and grouped by families.

FiveAP2/EREPB domain containing TFs expressed in the meristematic root, including *BABY BOOM1 *(*BBM*) (Mtr.21627.1.S1_at) and two with described Arabidopsis orthologs. Mtr.23155.1.S1_at is an ortholog to *PLETHORA1 *and Mtr.45360.1.S1_at is an ortholog of *Arabidopsis ANTIGUMENTA-LIKE 5*. Tandem AP2 domain transcription factors are strongly associated with plant development, and PLETHORA and BABY BOOM with root development where they have recently been shown to be dose-dependent regulators of root stem cell identity and maintenance [55]. The accumulation of these transcripts in the *Medicago *root meristem and absence of expression in the differentiated root is consistent with these findings.

Auxin is key regulator of plant gene expression and the AUX/IAA and ARF TFs are important regulators of auxin response. AUX/IAA TFs repress the expression of auxin activated genes until they are degraded by the SCF^TIR1 ^E3 ubiquitin ligase complex in the presence of IAA. ARFs can activate or repress transcription in the presence of auxin by binding to auxin-response elements. Two AUX/IAA transcripts are highly expressed in the meristem, Mtr.22904.1.S1.at and Mtr.16803.1.S1.at that are orthologous to *Arabidopsis IAA33 *and *IAA30 *respectively; neither has a described functional role in roots, but *IAA30 *is expressed in the root QC and during embryo maturation [12,56]. Two ARFs are highly expressed in RAM of *M. truncatula*, Mtr.13650.1.S1_at and Mtr.39233.S1_at share similarity with the open reading frames of *Arabidopsis ARF8 *and *ARF10 *respectively. In *Arabidopsis ARF 8 *has been shown to be involved in controlling the level of free IAA via a negative feedback loop by regulating the expression of IAA conjugating GH3 enzymes [57]. *ARF10 *has been shown to restrict the size of the stem cell niche in the distil root causing the differentiation of root cap cells, it is regulated by both IAA and miR160 [58].

Transcription factors with no similarity to those with a described role in meristems were also screened including two basic helix-loop-helix domain containing genes (Mtr.49495.1.S1_at, Mtr.24270.1.S1_s_at), a PLATZ domain (Mtr.46508.1.S1_at) and AT HOOK domain (Mtr.20966.1.S1_at) TFs; PLATZ and AT HOOK containing TFs have not previously been shown to be expressed in plant stem cells. Quantitative RT-PCR confirmed that all these TFs accumulate significantly in the meristem (Table 1).

Although no TF families are significantly over-represented in the non-meristem, it is of interest that two GRAS domain TFs are significantly expressed in this section. Several GRAS domain containing TFs are known to have roles in the root, the best characterized are the *Arabidopsis *GRAS genes *SCARECROW *(*SCR*) which in combination with *SHORT ROOT *are required for the specification of the QC and endodermis [59], two GRAS domain containing TFs have also been shown to be required for the establishment of nodulation in *Lotus japonicus *[60]. Our analysis showed the accumulation of GRAS transcript (Mtr.1484.1.S1_at) in the non-meristem. It is an ortholog of *Arabidopsis *gene *LATERAL SUPPRESSOR *(*LAS*); *LAS *has been show to suppress the formation of auxiliary meristems in *Arabidopsis *shoots, the mRNA was shown to also accumulate in roots but the effect of the mutation on lateral root development was not described [61]. It may have a role in inhibition of lateral root initiation.

## Conclusion

We have described differences between the root meristem and non-meristematic transcriptomes. Notably they include significant variations in carbon and flavonoid metabolism, auxin and cytokinin signalling, cell to cell communication and gene regulation. This data will facilitate the mapping of regulatory and metabolic networks involved in root meristem establishment and maintenance, and may lead to a better understanding of root stem cells in *M. truncatula *and other species with open meristem organisation where different mechanisms must operate to control meristem size and cell fate than those that operate in *Arabidopsis*.

## Methods

### Plant material

Seeds of *M. truncatula *accession A17 were scarified, surface-sterilised with 6% hypochlorite solution and washed 7 times with sterile distilled water. Seeds were germinated on nitrogen-free Fåhraeus medium on Petri plates in the dark for 24 to 30 hours. To provide intact primary roots for sectioning, germinated seeds that lacked any visible signs of microbial contamination were transferred to new Petri plates, 14 to 16 seedlings per plate, and grown for a further 3 days in a growth chamber until the roots had reached a length of 3 to 4 cm and before lateral roots emerged. At least 150 plants were required per RNA extraction.

Plates were kept vertically and the bottom half of each plate was sealed with Nescofilm R. Light was kept from the roots by the insertion of a black sheet between the plates during incubation. An aluminium foil spacer was placed under the lid of the Petri dish to allow gas exchange. Plates were incubated in a growth chamber at 20°C over a 16 hour photoperiod and a photon flux density of 100 mmol m^-2 ^s^-1 ^and 86% relative humidity.

To compare meristematic and non-meristematic root tissues, root sections were harvested from 3 day old plants. Tissue 3 mm from the root tip which contains meristematic cells and a further 1 cm section from the root containing non-meristematic cells were collected. All harvested plant materials were immediately frozen in liquid nitrogen and stored at -80°C.

For structural analysis, roots were fixed in phosphate buffer and glutaraldehyde, taken through an ethanol dehydration series then embedded in araldite [62]. Sections 1.5 μM thick were cut using a Leica Ultracut, stained with toluidine blue and viewed using a Zeiss Axioskop.

### RNA isolation, hybridization and data pre-processing

Total RNA was extracted and purified from plant tissues using the Qiagen RNeasy plant mini kit (Qiagen, Valencia, CA, USA). Total RNA was quantified using a NanoDrop ND-1000 Spectrophotometer; RNA with an absorbance A_260_/A_280_ratio > 2.0 was quality tested using the Agilent 2100 Bioanalyzer.

Preparation of cRNA, hybridization, and scanning of the Test3 arrays and *Medicago *GeneChip^® ^were performed according to the manufacturer's protocol (Affymetrix, Santa Clara, CA, USA) (at the Biomolecular Resource Facility, JCSMR, ANU). Briefly, double-stranded cDNA was synthesized from 5 to 8 μg of each RNA sample via oligo T_7_-(dT)_24 _primer-mediated reverse transcription. Biotin-labelled cRNA was generated using the Enzo BioArray kit (Affymetrix), purified using RNeasy spin columns (Qiagen), and then quantified by spectrophotometer. Fifteen to 20 μg of each biotin-labelled fragmented cRNA sample was used to prepare 300 μL of hybridization mixture. Aliquots of each sample (100 μL) were hybridized onto Test3 arrays to check the quality of the samples prior to hybridization (200 μL) onto the *Medicago *genome arrays. The arrays were washed with optimized wash protocols, stained with strepdavidin/phycoerythrin followed by antibody amplification, and scanned with the Agilent GeneArray Scanner (Affymetrix).

To remove certain systematic biases from the microarray, the raw Affymetrix data (.cel files) were normalized with the GCRMA (GC content – Robust Multi-Array Average) algorithm (ver. 2.2.0) including quantile normalization and variance stabilisation [63], using the *affy *package of the bioconductor software [64]. The normalized average of the replicates was then log transformed in base 2 to reduce the proportional relationship between random error and signal intensity. Differentially expressed probe sets were identified by evaluating the log_2 _ratio between the two conditions associated to a standard *t*-test [65]. All probe sets that differed by more than a two-fold difference with a *t*-test p ≤ 0.05 were considered to be differentially expressed.

### Genome array data analysis

Functional categories significantly associated (p ≤ 0.05, adjusted using the Bonferroni correction) with the up- and down-regulated sequences were identified using GeneBins, a database that provides a hierarchical functional classification modelled on the KEGG ontology [66] of probe set sequences represented on Affymetrix arrays [67]. We used PathExpress [68], a web-based tool based on the KEGG Ligand database [69], to detect whether probe sets associated with a metabolic pathway or sub-pathway were statistically over-represented in the differentially expressed sets of sequences (p ≤ 0.05).

In addition, probe sets of the Affymetrix *Medicago *genome array were assigned to gene families described in the TAIR database (Rhee *et al*., 2003) and to transcription factor families provided by the Database of Arabidopsis Transcription Factors [30] based on their sequence similarity with *Arabidopsis thaliana *proteins. BLASTXx [70] was used to find the best match (E ≤ 10^-8^) for the sequences representing each probe set (i.e. sequences derived from the most 5' to the most 3' probe in the public UniGene cluster). The differentially expressed sets of sequences were compared to the composition of each gene family to identify if a certain category was statistically over-represented. For each test, a *P*-value, representing the probability that the intersection of the list of up- or down-regulated probe sets with the list of probe sets belonging to the given gene family occurs by chance, was calculated using the hypergeometric distribution [71].

Sequences of interest were analysed using BLAST and multiple sequence alignments to identify genes and proteins with sequence similarity from *Arabidopsis*. To identify orthologs in *Arabidopsis *AffyTrees was used [72,73], AffyTrees automatically detects sequence orthologs based on phylogenetic trees.

### Quantitative Real-Time PCR

Total RNA was isolated from three biological repeats of tissue harvested from *M. truncatula *as described above using the Qiagen RNeasy MINI kit (Qiagen). The RNeasy kit protocol was modified to incorporate a DNase treatment using the DNase spin columns (Qiagen). cDNA synthesis was performed with SuperScript™ III reverse transcriptase (Invitrogen) using 2 μg total RNA for each sample using oligo (dT18) primers. For the no reverse transcriptase control, water was added instead of SuperScript III. For the real-time reverse transcription polymerase chain reaction (RT-PCR), gene specific primers were designed using Primer Express software (Applied Biosystems, Foster City, CA, USA) and ordered from Sigma Genosys. The PCR was carried out in a total volume of 10 μL containing 0.3 μM of each primer, 1 × SYBR green PCR master mix (Applied Biosystems). Reactions were amplified as follows: 95°C for 10 min, then 40 cycles of 95°C for 15 sec, 60°C for 1.5 min. Amplifications were performed in 384-well clear optical reaction plates (Applied Biosystems) with an ABI PRISM 7900 Sequence Detection System (at the Biomolecular Resource Facility, JCSMR, ANU) using version SDS 2.2.2 software (Applied Biosystems) to analyse raw data. The absence of genomic DNA and non-specific by-products of the PCR amplification was confirmed by analysis of dissociation curves and agarose gel electrophoresis using 3% agarose gels stained with 0.5 μg mL^-1 ^ethidium bromide. Normalisation was done as described by Searle *et al*. [74]; against the MtUBQ10 gene by calculating differences between the *C*_T _of the target gene and the *C*_T _of Ubiquitin 10 and relative gene expression levels were calculated.

The transcripts whose expression levels were verified by quantitative RT-PCR were as follows:

Mtr.20966.1.S1_at (AT HOOK), FP, 5'-TCG AGT AAT CGG AGG TGC TGT T-3', RP, 5'-ATG AAG CTC CCC ACT ACA ATT TG-3'.

Mtr.16722.1.S1_at (DVL like), FP, 5'-TCA AAA CTT GAA GTA CAA GCA AGG A-3', RP, 5'-CAC AAC GAC GAA CGA TGT AGA GA-3'.

Mtr.46508.1.S1_at (PLATZ), FP, 5'-TTG CAC TAG TAT TTG TCC TCA TTG C-3', RP, 5'-TGA TAA ACA TAA CGA CGA ACT TGA AGA-3'.

Mtr.32712.1.S1_at (LOB), FP, 5'-TTG CAC TAG TAT TTG TCC TCA TTG C-3', RP, 5'-AGG GCA TTC CTC AGC ACA TC-3'.

Mtr.49764.1.S1_at (MtPIN9), FP, 5'-CCA CTC TTC GCC TTC GAG TT-3', RP, 5'-TGT CCG CGC CTA TAA ATA AGA AGT-3'.

Mtr.49495.1.S1_at (bHLH), FP, 5'-GTC TCC AAG TTG CAG CAA CTT CT-3', RP, 5'-GCA ATA CCC TCG AAG CTG AAA-3'.

Mtr.21627.1.S1_at (AP2/EREBP), FP, 5'-TTG TTG CAT GAA TAG ATG ATT TGA GA-3', RP, 5'-CCT TCT TCA AGA TAC ATG CCA ATG-3'.

Mtr.24270.1.S1_s_at (bHLH), FP, 5'-GAC CAA AGC TGC CAT AGC TGA T-3', RP, 5'-GTC CTG GTC TTG TCC TAG TGA GAA TT-3'.

Mtr.39218.1.S1_at (GIF), FP, 5'-GAG GAA GGG ACA CGC AGT TC-3', RP, 5'-TCT TGT CTC TCA CTC TGC AAC GTT-3'.

Mtr.50542.1.S1_at (GFR), FP, 5'-AGG CAC TGA CAT CAA GTC AAC AA-3', RP, 5'-CTA GCC AGG AAT CTG TGT TCT TTG-3'.

The internal control gene was UBQ10 (TC100142), FP, 5'-GAA CTT GTT GCA TGG GTC TTG A-3', RP, 5'-CAT TAA GTT TGA CAA AGA GAA AGA GAC AGA-3'.

qRT-PCR for each gene was done on three biological replicates with duplicates for each biological replicate and no RT control. The relative transcript level was determined for each sample, normalised using the UBQ10 cDNA level, and averaged over three replicates and log transformed in base 2 to reduce the proportional relationship between random error and signal intensity. Significant variation from the internal control was determined using a *t*-test where p ≤ 0.05 was considered to be differentially expressed.

## Authors' contributions

PH conducted all experiments and drafted the manuscript. NG and GFW performed statistical and bioinformatics analysis. BGR and NI participated in the design of the study and assisted with manuscript preparation.

## Supplementary Material

Additional file 1 Microarry expression ratios for the *M. truncatula *root meristem (RM) and non-meristem (NMR). All quantitative data is expressed as log_2_(meristem:non-meristem) expression ratios. Click here for file

Additional file 2 Gene family classification for transcripts ≥2.0 fold differentially expressed. Click here for file

Additional file 3 Transcription factors ≥2.0 fold differentially expressed, as predicted by homology relationship based on members of Database of Arabidopsis Transcription Factors. Click here for file
